# Does Health Literacy of Hemodialyzed Patients Predict the Type of Their Vascular Access? A Cross-Sectional Study on Slovak Hemodialyzed Population

**DOI:** 10.3390/ijerph17020675

**Published:** 2020-01-20

**Authors:** Martina Zavacka, Ivana Skoumalova, Andrea Madarasova Geckova, Jaroslav Rosenberger, Peter Zavacky, Jana Pobehova, Maria Majernikova

**Affiliations:** 1 Vascular Surgery Clinic, Faculty of Medicine, P. J. Safarik University, Trieda SNP 1, 040 11 Kosice, Slovakia; martinazavacka80@gmail.com (M.Z.); jana.pastvova@post.cz (J.P.); 2 Department of Health Psychology and Research Methodology, Faculty of Medicine, P. J. Safarik University, Trieda SNP 1, 040 11 Kosice, Slovakia; andrea.geckova@upjs.sk (A.M.G.); jaroslav.rosenberger@upjs.sk (J.R.); 3 Graduate School Kosice Institute for Society and Health, P. J. Safarik University, Trieda SNP 1, 040 11 Kosice, Slovakia; 4 Olomouc University Social Health Institute, Palacky University in Olomouc, Univerzitni 22, 771 11 Olomouc, Czech Republic; 5 FMC—Dialysis Services Slovakia, Trieda SNP 1, 040 11 Kosice, Slovakia; mamajern@gmail.com; 6 II. Internal Clinic, Faculty of Medicine, P. J. Safarik University, Trieda SNP 1, 040 11 Kosice, Slovakia; 7 I. Surgery Clinic, Faculty of Medicine, P. J. Safarik University, Trieda SNP 1, 040 11 Kosice, Slovakia; peterzavacky963@gmail.com

**Keywords:** health literacy, vascular access, central venous catheter, arteriovenous fistula, dialyzed patients, chronic kidney disease (CKD-5)

## Abstract

Effective vascular access (VA) is an essential condition for providing hemodialysis, affecting patients’ health outcomes. We aim to explore how health literacy (HL) as a non-clinical factor is associated with the decision-making process regarding VA type selection. Using data from 20 dialysis centers across Slovakia (*n* = 542, mean age = 63.6, males = 60.7%), the association of HL with type of VA (arteriovenous fistula (AVF) vs. central venous catheter (CVC)) was analyzed using a logistic regression model adjusted for sociodemographic characteristics and comorbidity. Sociodemographic data and data on nine domains of HL were collected by questionnaire. Data on VA and comorbidity were obtained from a medical records. Patients with a greater ability to engage with healthcare providers (odds ratio (OR): 1.34; 95% confidence interval (CI): 1.00–1.78), those with a better ability to navigate the healthcare system (OR: 1.41; 95% CI: 1.08–1.85), those more able to find good health information (OR: 1.52; 95% CI: 1.15–2.03), and those who understand it well enough to know what to do (OR: 1.52; 95% CI: 1.12–2.06) are more likely to have AVF. Patients’ HL is associated with the type of VA; therefore, it should be considered in the decision-making process regarding the selection of the type of VA, thereby informing strategies for improving patients’ HL and doctor–patient communication.

## 1. Introduction

The prevalence of chronic kidney disease (CKD) increased rapidly over the last few decades, and it is a major public health problem, with a global prevalence of approximately 11%–15% and with a prevalence of 0.1% for end-stage renal disease (ESRD) patients [1], who require hemodialysis treatment or kidney transplantation. The life expectancy of dialyzed patients increased due to better disease management and more effective dialysis techniques, but this places increased demands on the type of vascular access (VA), which is able to work without the risk of thrombosis, inflammation, and other complications over the long term [2,3].

Hemodialysis requires one of the three types of VA—arteriovenous fistula (AVF), arteriovenous graft, or central venous catheter (CVC). AVF is considered to be the best option due to having the lowest association with patient morbidity and mortality [4,5,6,7], as well as lower rates of hospitalization [8], even in the older population of ESRD patients [4,9], and it is strongly recommended by clinical practice guidelines for VA [3]. A well-functioning VA is essential for effective hemodialysis treatment, but the selection of the VA type requires taking into consideration many factors that affect the possible access failure, such as the patient’s demographic factors, the patient’s adherence to fistula care, and cooperation between healthcare professionals and the patient [2,5]. Several modifiable factors are associated with the selected type of VA, such as the patients’ preference [10,11] and health literacy (HL) [12].

HL is defined as “the cognitive and social skills which determine the motivation and ability of individuals to gain access to, understand, and use information in ways which promote and maintain good health” [13]. Low HL is common in dialyzed patients and is associated with worse health outcomes [14,15], which may limit the implementation of AVF; however, the association of HL and the type of VA was not sufficiently explored to this date. We hypothesize that higher HL will be associated with AVF in dialyzed patients. The aim of this study is, therefore, to explore the association of HL and the type of VA of dialyzed patients using nationally representative data from 20 dialysis centers across Slovakia, while taking into account age, gender, education, and comorbidity of patients.

## 2. Materials and Methods

### 2.1. Sample and Procedure

**Figure 1:**
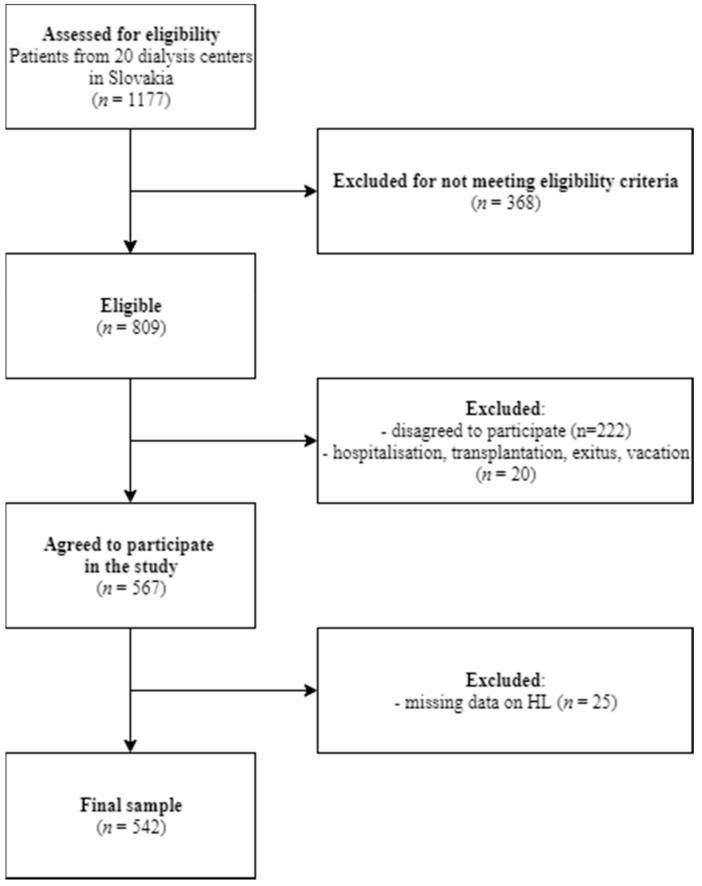
Flow chart related to data collection.

The study was observational with a cross-sectional design. Data were collected in 20 dialysis clinics in Slovakia from January to November 2018. Cluster sampling was used to recruit the sample (Figure 1). We included patients older than 18 years with a diagnosis of CKD-5 and those on hemodialysis treatment for at least for 90 days. We excluded those who were not able to fill in the questionnaire for various reasons (dementia, mental retardation, psychiatric diagnosis, inability to speak and read in Slovak language, and those with acute severe intercurrent illness), similar to other studies concerning dialyzed patients [16,17].

Questionnaire data were obtained from patients by filling in an online questionnaire on tablets during their dialysis session. Clinical data were obtained from a medical database—European Clinical Database (EuClid5)—closest to the time of completion of the questionnaires. All patients signed an informed consent prior to the data collection.

### 2.2. Ethics

The study was approved by the Ethics Committee of the Faculty of Medicine, Pavol Jozef Safarik University (15N/2017) and the Ethics Committee of FMC-Dialysis services (23 November 2017), reflecting the ethical standards as laid out in the 1964 Declaration of Helsinki and its later amendments.

### 2.3. Measures

Data on vascular access were obtained from the medical database (EuClid5) and dichotomized as zero (central venous catheter) and one (arteriovenous fistula).

Data on health literacy (HL) were obtained using the Slovak version of the Health Literacy Questionnaire (HLQ) [18,19]. This multidimensional tool consists of nine distinct domains of health literacy which are related to the way how patients access, understand, and use information about health. A higher score in a particular domain means a higher level of health literacy. The mean score of each domain was used for analyses. The instrument is divided into two parts, each with different instructions and answering options. The mean score for each domain was used for analysis. In the first part, which covers domains 1–5, respondents are asked to what extent they agree with the statements. Answering options include four response categories: 1—strongly agree, 2—agree, 3—disagree, and 4—strongly disagree. Domains 1–5 have a mean score ranging from 1–4. These domains are (1) feeling understood and supported by a healthcare provider, (2) having sufficient information to manage my health, (3) actively managing my health, (4) social support for health, and (5) appraisal of health information. In the second part, which covers domains 6–9, respondents are asked how easy or difficult certain tasks are for them. Answering options include five response categories: 1—cannot do, 2—very difficult, 3—quite difficult, 4—quite easy, and 5—very easy. Domains 6–9 have a mean score ranging from 1–5. These domains are (6) ability to actively engage with healthcare providers, (7) navigating the healthcare system, (8) ability to find good health information, and (9) understanding health information well enough to know what to do.

Sociodemographic data were measured by a questionnaire and included age (used as a continuous variable), gender, and the educational level (used as a dichotomized variable: lower education (patients with elementary education and apprenticeship) vs. higher education (patients with secondary education and university)).

Data on comorbidity were obtained from medical records. The Charlson comorbidity index (CCI, age-adjusted continuous variable) which has a score ranging from 0–33, with a higher value meaning higher comorbidity [20,21], was used as a continuous variable. The data on the presence of diabetes mellitus (type 1 and 2) were used as a dichotomized variable.

### 2.4. Statistical Analyses

Firstly, we assessed the sociodemographic characteristics and the type of vascular access in the study sample. Secondly, we explored which of the nine domains of health literacy were associated with the type of vascular access. For statistical analysis, we used logistic regressions adjusted for age, gender, education, Charlson comorbidity index, and the presence of diabetes mellitus (type 1 and 2). Odds ratios (OR) with 95% confidence intervals (CI) were used for each type of vascular access with a *p*-value of 0.05 assumed for statistical significance.

All statistical analyses were performed using SPSS v. 23.0 for Windows [22].

## 3. Results

### 3.1. Baseline Characteristics

We included 567 dialyzed patients (70% of those approached), and 25 patients were excluded due to missing data on HL. The final sample consisted of 542 patients (Table 1).

### 3.2. Associations of HL and the Type of VA

Table 2 shows the association between HL domains and the type of VA adjusted for age, gender, education, CCI, and diabetes mellitus. Four domains were significantly associated with the type of VA. Patients with greater ability to engage with healthcare providers (HLQ6; OR: 1.34, 95% CI: 1.00–1.78), those with a better ability to navigate the healthcare system (HLQ7; OR: 1.41, 95% CI: 1.08–1.85), those more able to find good health information (HLQ8; OR: 1.52, 95% CI: 1.15–2.03), and those who understand it well enough to know what to do (HLQ9; OR: 1.52. 95% CI: 1.12–2.06) are more likely to have AVF as their VA.

## 4. Discussion

In our study, we explored the association of HL and the type of VA adjusted for age, gender, education, and comorbidity. We found that patients with greater ability to engage with healthcare providers, those with better ability to navigate the healthcare system, those more able to find good health information, and those who understand it well enough to know what to do are more likely to have AVF as their VA. Cavanaugh et al. [23] brought similar findings, whereby dialyzed patients with limited HL were more likely to have CVC. Mazarova et al. [12] focused on functional HL in dialyzed patients and their choice of the type of VA, and they found that patients with low HL were more likely to prefer CVC. They concluded that this is due to a lack of education and information for patients from healthcare providers needed for informed decision-making about their VA. This is in line with our findings, where patients able to find information, able to engage and communicate with healthcare providers, and able to understand information about health so they can be adherent with their choices showed a higher chance for AVF as their VA. On the contrary, Green at al. [18] did not find HL to be an independent predictor of the type of VA. All studies to date concerning this topic and mentioned above used a one-dimensional measurement tool to access HL.

Low HL is associated with worse health outcomes in dialyzed patients [14], and high morbidity may be considered as a limitation for AVF when making decisions about the type of VA. According to Ravani et al. [5], healthier patients are more likely to use an AVF for hemodialysis. In our study, after also adjusting for comorbidities, the association of HL and the type of VA still remained significant.

Not just patients might differ in health literacy; physicians might also differ in their capacity to work with patients with low health literacy, and healthcare units might differ in their responsiveness to patients with low health literacy [24]. Different healthcare units might apply different rules for decision-making related to vascular access. Moreover, a physician’s experience with patients´ communication skills might play a significant role in decision-making. Further research on the decision process might indeed help us to understand better factors and mechanisms, which in the end lead to inequality in healthcare.

Our conclusion on the association of high patient’s health literacy with a higher chance of having AVF, which is associated with better health outcomes in ESRD patients, might be generalized to patients in most industrialized countries, as dialysis clinics follow guidelines and might differ in their responsiveness mainly due to different training of medical staff. Therefore, low health literacy remains a risk factor with regard to inequality in access to healthcare.

### 4.1. Strengths and Limitations

The major strength of our study is the representativeness of our sample, which consists of approximately 20% of the dialysis population in Slovakia; therefore, we consider the results of our study as generalizable. Another strength is the use of a multidimensional measurement of HL, which allowed us to identify potential modifiable areas of HL in dialyzed patients regarding HL [25,26].

The major limitation of our study is the cross-sectional design; therefore, no interpretations on causal mechanisms are possible.

### 4.2. Implications

Our findings show that health literacy may be an important non-clinical factor playing a role in the decision-making process about the type of VA. AVF is considered to be the first choice in dialyzed patients due to its association with lower morbidity and mortality in comparison with CVC; therefore, all clinically suitable patients should have access to this type of VA. To ensure this, strategies to improve HL in dialyzed patients may be helpful to increase their chances of having AVF. It is also important to ensure that healthcare professionals provide sufficient information and education to avoid refusal of AVF by patients, as well as information regarding proper adherence to AVF care. They should also pay attention to varying HL needs and limitations in dialyzed patients and to patients’ fears and misunderstandings regarding the information they get. Assessing patients’ HL should become a standard routine in healthcare in earlier stages of CKD to prevent the implementation of CVC where not necessary.

Future research should focus on the association of various factors connected with the decision-making process about the type of VA, e.g., reasons for patients’ refusal of AVF, as well as on the capacities of healthcare professionals to provide adequate information about the risks and benefits of AVF in comparison with CVC.

## 5. Conclusions

We found that higher HL in dialyzed patients is associated with a higher chance of having AVF, which is associated with better health outcomes in ESRD patients. As comorbidity was considered, it may indicate that HL operates as a non-clinical factor in decision-making when the type of VA is selected.

## Figures and Tables

**Table 1:** Characteristics of the sample: age, gender, education, type of vascular access (VA), and comorbidity; frequencies or means (*n* = 542).

Characteristics of the Sample	M	SD	*n*	%
*Demographic characteristics*				
Age	63.6	14.1		
Male			329	60.7
Lower education			266	49.1
*Type of Vascular access*				
CVC			143	26.8
AVF			390	73.2
*Comorbidity*				
CCI ^1^	6.5	2.9		
Diabetes mellitus			183	34.1

^1^ The range of scores was 2–23. M—mean; CVC—central venous catheter; AVF—arteriovenous fistula; CCI—Charlson comorbidity index.

**Table 2:** The associations between health literacy domains and the type of vascular access. Logistic regression model adjusted for age, gender, education, CCI, and diabetes mellitus (*n* = 542).

HLQ Domain	HLQ Domain title	Type of Vascular Access OR (95% CI)
HLQ1	Feeling understood and supported by a healthcare provider	1.19 (0.77–1.83)
HLQ2	Having sufficient information to manage health	1.07 (0.70–1.63)
HLQ3	Actively managing my health	0.96 (0.61–1.51)
HLQ4	Social support for health	1.22 (0.80–1.86)
HLQ5	Appraisal of health information	0.99 (0.68–1.44)
HLQ6	Ability to actively engage with healthcare providers	1.34 (1.00–1.78) *
HLQ7	Navigating the healthcare system	1.41 (1.08–1.85) *
HLQ8	Ability to find good health information	1.52 (1.15–2.03) **
HLQ9	Understand health information well enough to know what to do	1.52 (1.12–2.06) **

* *p* < 0.05, ** *p* < 0.01. Missing cases due to missing data on the type of VA (nine cases). HLQ—Health Literacy Questionnaire; OR—odds ratio; CI—confidence interval.
